# Registry-derived stage (RD-Stage) for capturing stage at diagnosis for pancreatic carcinoma in Australia

**DOI:** 10.1371/journal.pone.0294443

**Published:** 2024-01-02

**Authors:** Sue M. Evans, Kris Ivanova, Danca Cossio, Charles H. C. Pilgrim, Daniel Croagh, John Zalcberg, Dalisay Giffard, Nikkitia Golobic, Bruno Di Muzio, Catriona McLean C, Kate McLean, Gregory C. Miller, Susanna Nicosia, Nick O’Rourke, Sumit Parikh, Richard Standish, Luc te Marvelde

**Affiliations:** 1 Cancer Council Victoria, Melbourne, Australia; 2 School of Public Health and Preventive Medicine, Monash University, Clayton, Australia; 3 Cancer Alliance Queensland, Woolloongabba, Australia; 4 Central Clinical School, Department of Surgery, The Alfred Hospital, Monash University, Clayton, Australia; 5 Department of Surgery, Monash Health and Monash University, Clayton, Australia; 6 Dept. of Medical Oncology, Alfred Health and School of Public Health and Preventive Medicine, Monash University, Clayton, Australia; 7 Department of Radiology, Alfred Hospital, Australia; 8 Department of Anatomical Pathology, Alfred Health, Australia; 9 Department of Diagnostic Imaging, Princess Alexandra Hospital, Australia; 10 Envoi Specialist Pathologists, Queensland Australia; 11 Faculty of Medicine, University of Queensland, Queensland Australia; 12 Department of Surgery, Royal Brisbane Hospital, Herston, Australia; 13 Dorevitch Pathology (Geelong), Australia; 14 School of Medicine, Deakin University, Australia; The University of Sydney, AUSTRALIA

## Abstract

**Introduction:**

Stage of pancreatic carcinoma at diagnosis is a strong prognostic indicator of morbidity and mortality, yet is poorly notified to population-based cancer registries (”cancer registries”). Registry-derived stage (RD-Stage) provides a method for cancer registries to use available data sources to compile and record stage in a consistent way. This project describes the development and validation of rules to capture RD-Stage (pancreatic carcinoma) and applies the rules to data currently captured in each Australian cancer registry.

**Materials and methods:**

Rules for deriving RD-stage (pancreatic carcinoma) were developed using the American Joint Commission on Cancer (AJCC) Staging Manual 8^th^ edition and endorsed by an Expert Working Group comprising specialists responsible for delivering care to patients diagnosed with pancreatic carcinoma, cancer registry epidemiologists and medical coders. Completeness of data fields required to calculate RD-Stage (pancreatic carcinoma) and an overall proportion of cases for whom RD stage could be assigned was assessed using data collected by each Australian cancer registry, for period 2018–2019. A validation study compared RD-Stage (pancreatic carcinoma) calculated by the Victorian Cancer Registry with clinical stage captured by the Upper Gastro-intestinal Cancer Registry (UGICR).

**Results:**

RD-Stage (pancreatic carcinoma) could not be calculated in 4/8 (50%) of cancer registries; one did not collect the required data elements while three used a staging system not compatible with RD-Stage requirements. Of the four cancer registries able to calculate RD-Stage, baseline completeness ranged from 9% to 76%. Validation of RD-Stage (pancreatic carcinoma) with UGICR data indicated that there was insufficient data available in VCR to stage 174/457 (38%) cases and that stage was unknown in 189/457 (41%) cases in the UGICR. Yet, where it could be derived, there was very good concordance at stage level (I, II, III, IV) between the two datasets. (95.2% concordance], Kendall’s coefficient = 0.92).

**Conclusion:**

There is a lack of standardisation of data elements and data sources available to cancer registries at a national level, resulting in poor capacity to currently capture RD-Stage (pancreatic carcinoma). RD-Stage provides an excellent tool to cancer registries to capture stage when data elements required to calculate it are available to cancer registries.

## Introduction

Cancer stage at diagnosis provides important information to guide treatment and to understand prognosis following a diagnosis of pancreatic carcinoma. It also supports cancer control activities and enables epidemiological and health services research to be undertaken to improve the management of cancer services for people diagnosed with pancreatic cancer.

The tumour-node-metastasis (TNM) classification system was first developed by French surgeon Pierre Denoix more than 70 years ago as a means of describing the anatomic extent of cancer [1]. In 1958, the first TNM recommendations were published for breast and laryngeal cancers by the Union for International Cancer Control (UICC), and by 1967, this had expanded to classify tumours from a further 23 body sites. In 1982, a single classification system was developed for solid tumours [2]. From 1987 onwards, both the UICC and the American Join Committee on Cancer (AJCC) produced cancer staging systems using TNM classification, which are nearly identical and both are currently in their 8^th^ edition [3, 4].

While the TNM classification system is the most frequently used, there are other staging classification systems. This includes one developed by the International Federation of Gynecology and Obstetrics (FIGO) for gynaecological cancers [5], which maps to the UICC and AJCC TNM classification systems. Other classification systems developed by the Surveillance, Epidemiology and End Results (SEER) program include Extent of Disease (EOD) and the more basic Summary Stage classification systems [6]. Summary Stage classifies tumours according to the theory of cancer growth into five categories of in situ, localized, regionalized, distant and unknown. A limitation of Summary Stage is that it is not possible to convert all TNM codes to a Summary Stage and conversely, Summary Stage cannot be converted to TNM codes or AJCC/UICC stage groups [7]. As such, its use has predominantly been limited to the cancer surveillance community [8]. Since 2018, cancer registries in the United States have transitioned to collecting Extent of Disease, which was designed to be compatible with the AJCC 8^th^ edition TNM classification system.

In the management of pancreatic carcinoma, the use of cross-sectional imaging has resulted in a system which classifies tumour not by the extent of the disease using traditional TNM, but by the ability of surgeons to resect the tumour (resectable, borderline resectable, unresectable [locally advanced] or unresectable/metastatic) [9]. This recognizes that the ability for surgeons to operate is impacted by the location of the tumour and its proximity to surrounding vascular structures and whether a reconstruction of the vasculature is possible, more than its size or lymph node involvement [10].

Capture of cancer stage information for pancreatic carcinoma by cancer registries is challenged not only by the many and varied cancer classification systems, but also by poor and varied recording of stage in medical records, and subsequent poor reporting by hospitals [11]. Stage is best determined and recorded at multidisciplinary team meetings, where imaging specialists, pathologists, radiation oncologists and surgeons review the tumour to determine a treatment plan based on the size, location and extent of spread of the tumour. Yet, stage is often poorly recorded at multidisciplinary team meetings, perhaps reflecting barriers impacting the overall quality of the meeting; including insufficient information available to make a determination, time constraints, poor teamwork, and case complexity [12, 13]. Most pancreatic carcinomas are staged in multidisciplinary team meetings using the clinical staging system as most cases present with advanced disease, precluding surgical removal and pathological stage assessment.

In recognition that routine collection of cancer stage at diagnosis was an identified national data gap in cancer knowledge, Cancer Australia through the Stage, Treatment and Recurrence (STaR) initiative has led work to improve capture of stage information. Collaborating with jurisdictional cancer registries, the Australasian Association of Cancer Registries (AACR) and state and territory health departments, the STaR project has led to AACR-endorsed rules for the collection of registry-derived (RD) stage for melanoma as well as prostate, lung, breast, and bowel cancer [14]. RD-Stage is defined as “*the best estimate of summary TNM stage at the time of diagnosis (or within 120 days of diagnosis and before primary cancer treatment) as derived by cancer registries from available data sources for use in population data analysis”* [15]. Available data sources included pathology reports and data provided by hospital administrative systems. This activity led to national stage data being published for the top 5 highest incident cancers in 2011 [16] and subsequent publication of 5-year survival statistics for these cancers [17]. Recently, RD-Stage (endometrial cancer) rules were developed and validated [18].

Because RD-Stage is calculated using only minimal data and does not consider other inputs such as imaging scans and clinical examination if they are not available, it was intended for epidemiological population-based analyses only and not to be used at a clinical level for individual patients [19].

The aim of this project was to develop rules to stage pancreatic carcinoma, assess capacity to report RD-Stage (pancreatic carcinoma) at a national level in Australia, and validate RD-Stage (pancreatic carcinoma) against stage of cancer at diagnosis reported by clinicians.

## Materials and methods

This study was undertaken between July 2021 and September 2022. The study was funded by Cancer Australia and involved developing and evaluating rules for two cancers- endometrial cancer and pancreatic cancer, with the endometrial cancer research discussed elsewhere [18]. A consent waiver was granted for the use of data collected from each PBCR to evaluate the initial reporting of RD-Stage (pancreatic cancer) and for subsequent validation work using data from the Upper Gastro-intestinal Cancer Registry (UGICR). Consent was secured from the participants in the Expert Working Group. This study was approved as a project under National Mutual Acceptance by the Monash Health Human Research Ethics Committee (HREC/76860/MonH-2021-272807), the South Australian Department of Health and Wellbeing (SA GEMS 2022/SSA00137) and the NSW Population Health Research Ethics Committee (2022/ETH00558).

### Development of staging rules

Rules for deriving RD-Stage (pancreatic carcinoma) were developed using the TNM Staging System (8^th^ Edition) [4]. The TNM system was used because it provided more granularity than SEER, is the most commonly used classification system globally and mapping work had already been undertaken to enable SEER classifications to be aligned with TNM [11]. While resectability has been compared with TNM stages to examine survival [9], rules were developed using the AJCC stage descriptors which consider only the location and size of the tumour and not whether it was considered resectable or not.

An Expert Working Group was assembled comprising an epidemiologist, clinical coding consultants (n = 4), consultant radiologists (n = 2), consultant pathologists (n = 4), a medical oncologist, two hepatobiliary and one general surgeon, and a statistical analyst. Draft rules were written and distributed in advance of the Working Group meeting with instruction to review the histology codes to be included in the model, assess the rules for each tumour (T)-, node (N)- and metastasis (M)- category, determine the time point up to which diagnostic stage can be measured and review diagnostic and treatment pathways used to assist medical coders in coding and following up on potentially missing information. The meeting was held via videoconference over two hours, with correspondence occurring via email after the meeting. A second meeting discussed unresolved issues and reached consensus on the rules. The Expert Working Group agreed that, where possible, cancer registries should also assess their ability to record resectability status. The group agreed to use the rules published by the National Comprehensive Cancer Network (NCCN) as the reference for recording resectability status [20]. These rules rely on use of imaging, with an imaging protocol and templated report to describe the required characteristics. NCCN recommends that the imaging is assessed by a multidisciplinary team to determine the final status.

### Capture of pancreatic carcinoma stage at diagnosis by cancer registries

Following the development and endorsement of rules, cancer registries were requested to complete an Excel spreadsheet containing the data fields required to calculate RD-Stage (pancreatic carcinoma) for the years 2018–2019 to ascertain how well each Australian jurisdiction could calculate RD-Stage without requiring any intervention. Three jurisdictions (New South Wales, Tasmania, and Australian Capital Territory) routinely captured SEER Summary Stage information and did not collect the required T, N and M categories required to derive RD-Stage. This information was requested so that baseline completeness of both Degree of Spread and RD-Stage could be assessed and compared. Both Victoria and Queensland had access to hospital admitted episode data, enabling nodal and metastatic site codes (C77-C79) from the International Classification of Diseases- 10th edition Australian Modified (ICD-10AM) coding system to be used to identify cases with metastatic disease at diagnosis [21]. The Queensland cancer registry managed by Cancer Alliance Queensland, operates under the umbrella of a broader clinical governance partnership, with additional legislation that provides access to data held in admitted episode datasets, oncology information systems, public radiology databases, and multidisciplinary team meeting notes from public, and some private, healthcare facilities [22] Cancer Alliance Queensland used the RD-Stage rules to extract implicit statements of TNM stage and resectability classification from imaging reports for patients who had no other stage information available from the other sources.

#### Validation of rules

Two medical coders employed by the Victorian Cancer Registry were trained in abstracting information for RD-Stage calculation from pathology reports. Data was populated into an Excel spreadsheet in which a macro was written to automatically calculate RD-Stage. Coders entered data for cases with eligible histology codes diagnosed between 2018 and 2020. This was supplemented with hospital admitted episode data where ICD-10AM metastatic site codes C77-C79 were coded. To test the RD-Stage rules, a validation dataset was obtained from the Upper Gastro-intestinal Cancer Registry (UGICR). The UGICR was established in 2015 in accordance with the strategic principles outlined in the Australian Commission on Safety and Quality in Health Care’s framework for clinical quality registries [23]. It is operated by the Cancer Research Program in the School of Public Health and Preventive Medicine at Monash University. Data collection and handling methods have been previously reported [11]. In summary, the VCR provides the UGICR personnel with patient details to enable them to recruit patients to the registry. Stage information which is clearly recorded in the case record by medical staff is captured by trained research assistants.

A file containing patient details and pathological and clinical stage information for cases diagnosed between 2018 and 2020 was provided by UGICR, where deterministic matching was used to link the two datasets (using the VCR unique identifier provided to the UGICR for linkage purposes when patient details were first released by the VCR).

A manual medical record review was undertaken to independently assess the accuracy of stage data captured by UGICR. A random blinded sample of 5% of medical records was undertaken by a medical coding expert (KI).

## Results

### 1. Developing RD-Stage rules

The Expert Working Group advised excluding pancreatic neuroendocrine tumours (NET) from inclusion in rules and subsequent validation work, because they have a low incidence (approximately 2% of pancreatic tumours are NETs), are staged differently to other pancreatic tumours [4], and have different presentation and outcomes [24]. Histology and topography codes under the International Classification of Diseases for Oncology version 3 (ICDO-3) classification system included in the rules and a summary of the distribution of these histology and topography codes in the national statistics between 2018 and 2020 (other than Queensland for which data was not provided) is shown in Table 1.

**Table 1:** International Classification of Diseases for Oncology version 3 (ICDO-3) histology and topography codes and percentage distribution among all pancreatic carcinoma diagnoses in Australia between 2018 and 2020.

Eligible histology codes and description*		Percent distribution in pancreatic cancer diagnoses- 2018–2020†
8020/3	Undifferentiated carcinoma	0.3%
8035/3	Undifferentiated carcinoma with osteoclast-like giant cells	0
8441/3	Serous cystadenocarcinoma	0
8453/3	Intraductal papillary mucinous neoplasm with associated invasive carcinoma	1.0%
8470/3	Mucinous cystic neoplasm with associated invasive carcinoma	0.1%
8480/3	Mucinous carcinoma	1.0%
8490/3	Signet ring cell carcinoma	0.1%
8500/3	Ductal Adenocarcinoma	16.4%
8503/3	Intraductal tubulopapillary neoplasm with associated invasive carcinoma	0.1%
8510/3	Medullary carcinoma	0
8550/3	Acinar cell carcinoma	0.4%
8551/3	Acinar cell cystadenocarcinoma	0
8552/3	Mixed acinar-ductal carcinoma	0
8560/3	Adenosquamous carcinoma	0.9%
8576/3	Hepatoid carcinoma	0
8000/3	Malignant Neoplasm, NOS	16.5%
8010/3	Carcinoma, NOS distribution	9.6%
8140/3	Adenocarcinoma, NOS	53.5%
8481/3	Mucin-producing adenocarcinoma	0.1%
**Topography codes and description**		
C250	Head of pancreas	40.6%
C251	Body of pancreas	8.5%
C252	Tail of pancreas	9.2%
C253	Pancreatic duct	1.2%
C257	Other specified parts of pancreas	3.0%
C258	Overlapping lesion of pancreas	1.8%
C259	Pancreas, NOS	35.7%

*Histology codes refer to those recorded in the WHO Classification of Tumors and the International Classification of Diseases for Oncology (ICD-O)

† Data obtained from all Australian population-based cancer registries other than Queensland

The Expert Working Group recommended that RD-Stage calculation commence with calculating the TNM-M value, as the presence of distant metastasis negated the need to capture TNM-N or TNM-T categories. There was debate over whether M0 could be assumed if it was not explicitly recorded that the patient had no metastasis. Classification of MX (unknown) was eliminated from the 6^th^ and subsequent editions of the AJCC and UICC TNM staging systems. The AJCC/UICC coding rules state that “Unless there is clinical or pathological evidence of distant metastases, the patient should be classified as clinical M0 and denoted as cM0. It is not necessary to perform any imaging or invasive studies to categorise a patient as cM0, only a history or clinical examination are required. The TNM-M category must always be known and reported to assign a stage group” [16]. As such, the rules state that an M0 is assumed if there is no clinical or imaging evidence of metastasis or if no distant metastasis is recorded. Based on having multiple data sources to extract stage data, Cancer Alliance Queensland modified the definition of M0 to state that it was only appropriate to record an M0 if the patient had a subsequent admission more than 120 days after the date of diagnosis and that it did not include an ICD-10AM metastatic site code.

Following classification of metastatic disease, coding should proceed to the N-category and finally to the T-category. A category of X is recorded for both the TNM-N and TNM-T fields if relevant information is not available or cannot be assessed. The S1 File document accompanying this manuscript contains the RD-Stage (pancreatic carcinoma) rules for calculating each category and instruction on how this information should be amalgamated to define RD-Stage, and a diagram indicating the sources of information required by cancer registries to calculate RD-stage. These tools were developed to support medical coders in calculating RD-Stage (pancreatic carcinoma).

### 2. Completeness of data fields required to calculate RD-Stage by cancer registries

Table 2 provides an outline of the completeness of data fields required to calculate RD-stage across Australian jurisdictions in 2018 and 2019. RD-Stage completeness rates were highest in Queensland (76%) and Victoria (65%), with low rates recorded in Northern Territory (9%) and Western Australia (9%). Northern Territory only recorded stage data if it was explicitly documented on the pathology report, while Western Australia captured it for a small, targeted study. While South Australia could theoretically calculate RD-Stage, none of the data fields required to calculate RD-Stage it were populated in their cancer registry in 2018 and 2019. Degree of spread completeness ranged from 69% to 87%, across both years in New South Wales and Australian Capital Territory. Tasmania recorded 65% completeness in 2018 but had not completed coding in 2019 at the time this project was conducted.

**Table 2:** Completeness of selected data fields in 2018 and 2019 for pancreatic carcinoma, by jurisdiction.

	2018 and 2019 years combined							
Jurisdiction	VIC	QLD	WA	SA	NSW	TAS	ACT	NT
Number of cases (n)	1754	1266	612	295	2123	87	102	36
Data completeness: proportion of cases with data field available								
Lymph nodes taken	14%	8%	-	-	-	14%	-	-
Lymph nodes positive	14%	5%	-	-	-	14%	-	-
Staging basis	65%	76%	8%	-	-	-	-	14%
TNM-T	17%	19%	8%	-	-	-	-	59%
TNM-N	18%	19%	8%	-	-	-	-	14%
TNM-M	49%	15%	6%	-	-	-	-	-
Tumour size	17%	14%	-	-	-	28%	-	59%
Stage group	3%	14%	6%	-	-	-	-	-
Degree of spread (Tier 1) ^*†*^	-	-	-	-	84%	65%	72%	-
RD-Stage (Tier 2) *	65%	48%	9%	-	-	-	-	59%
RD-Stage (Tier 3) ^#^	-	76%	-	-	-	-	-	-

^†^Degree of spread (Tier 1) includes Pathology and hospital admitted episode data:

* RD-Stage (Tier 2) includes sources: Pathology (VIC, WA, NT, QLD) and hospital admitted episode data (VIC only)

^#^ RD-Stage (Tier 3) includes sources: Pathology, multidisciplinary team meeting data, hospital admitted episode data, oncology information systems and public radiology data

The addition of hospital admitted episode data and data from multidisciplinary team meeting, imaging, and oncology databases available in Queensland enabled stage to be calculated in 76% of cases for the 2018/19 years combined. To gather cancer stage information in Queensland, a clinical advisory group was established to develop a hierarchical approach to capture of stage data from multiple sources. They recommended that stage data should initially be sourced from multidisciplinary team meeting software, then pathology reports if available. If not found in these sources, it should be sought from the oncology information system, followed by hospital admission data, and finally from imaging reports. Imaging reports were ranked as the lowest source, due to the time required to manually review the reports. Cancer Alliance Queensland found that where stage was recorded in multidisciplinary team meeting notes, it was generally in relation to resectability rather than TNM stage. There was no evidence in imaging reports of the use of standard templates to capture tumour characteristics required to define resectability and cancer stage, such as tumour location, vascular and lymph node involvement, and encasement of nearby structures [25, 26]. The complexity and multiple dependencies that are assessed in defining resectability status using imaging means that cancer registry staff are unlikely to be able to determine resectability status from the imaging report.

### Validation of RD-Stage

For the 2018–2020 years, there were 2,612 pancreatic incident cases recorded in the VCR. Of these, 1216/2612 (47%) reported TNM = M1 (metastatic disease) at diagnosis and 64/2612 (2%) had stage reported by the health service. When linked with the subset of 457 cases available in the UGICR, stage was available for 283 (62%) cases in the VCR and 260 (57%) cases in the UGICR. Direct comparison was available for 229 cases (Table 3).

**Table 3:** Stage distribution for cases reported in the Victorian Cancer Registry (VCR) and the Upper Gastro-intestinal Cancer Registry (UGICR).

Stage group	VCR-RD stage	UGICR
**Stage-total**	**283**	**260**
*Stage 1*	*25*	*23*
*Stage 2*	*26*	*23*
*Stage 3*	*19*	*18*
*Stage 4*	*213*	*196*
**Stage Unknown**	**165**	**189**
**Stage post neoadjuvant therapy**	**9**	**8**
**TOTAL**	**457**	**457**
**Total number of patients for Stage Validation (n)**	**229**	
**Equal stage distribution**	**218/229 (95.2%)**	
**Kendall’s coefficient of concordance**	**0.92; p < 0.05**	

There were 165 (36%) of cases in VCR which were unable to be staged because no details were provided on either the pathology or hospital report or the death certificate to enable stage to be derived, and there were 189 (41%) cases where stage was unable to be ascertained in the UGICR data. There were 31 cases staged in UGICR and not in the VCR and 47 cases in the VCR but not in the UGICR. There was agreement in stage distribution for 218 of 229 cases (95.2%) with significant Kendall’s coefficient of concordance of 0.92 (p<0.05), indicating a very good level of agreement [27]. There was unequal stage distribution in 11 cases, of which VCR under-reported the stage in six cases and over-reported in five cases compared to the UGICR. (Table 4)

**Table 4:** Distribution of stage in the Victorian Cancer Registry (VCR) and Upper Gastro-intestinal Cancer Registry (UGICR).

RD-Stage	UGICR Stage			
	Stage 1	Stage 2	Stage 3	Stage 4
**Stage 1 **	15	1		1
**Stage 2 **		16	4	
**Stage 3 **	2		8	
**Stage 4 **	2		1	179

The random audit of 23 cases (5% of records at the hospital) found no discrepancies in the reporting of stage. All UGICR cases with reported resectability status “Unresectable, (metastatic disease)” were accepted as TNM stage IV. There were 31 cases where RD stage could not be calculated.

An audit of the 31 cases where RD-stage could not be captured by the VCR indicated that 14 were diagnosed from clinical data not available to the VCR, 6 had missing surgical pathology, 7 did not have a distant metastasis reported and 4 did not have locally advanced cancer reported to the VCR.

While 47 cases recorded in the UGICR did not capture stage, resectability was recorded on 32 (68%) of these cases. Of these 47 cases, 22 could not be staged because data known to the VCR was not available to the UGICR, 21 did not record available pathology information and 4 did not capture locally advanced disease recorded in the medical record.

## Discussion

This study was undertaken to obtain a baseline snapshot of current capacity at a national level to capture RD-Stage (pancreatic carcinoma) by cancer registries. It shows that capture of standardised cancer stage at diagnosis at a national level in Australia remains an aspirational goal, as there are currently inconsistencies in both the type of data captured and the data sources used to collect it. New South Wales, Australian Capital Territory and Tasmania use the SEER Summary Stage which captures ‘degree of spread’ to indicate cancer stage at diagnosis. This study used the AJCC TNM classification system to develop rules for staging pancreatic carcinoma because it was the most used among the clinical community. However, the Expert Working Group acknowledged that resectability was a more common language used by radiologists and surgeons to stage pancreatic carcinoma. When data fields required to derive RD-Stage were captured by medical coders, this achieved a high level of concordance (>95%) with stage recorded by clinicians in medical records. However, RD-Stage could only be calculated for two in three patients because necessary data fields were not available to cancer registries, or the patient received neoadjuvant therapy prior to data being made available to the cancer registry. Audit of cases for which stage could not be captured by the VCR have also identified opportunities for improvement. More than 94% of pathology notifications are transmitted automatically from laboratories [28], and the finding that some were not available to medical coders when calculating RD-Stage indicates a need to assess case finding criteria and processing of paper notifications by VCR.

This study has demonstrated that capture of stage data for patients diagnosed with pancreatic carcinoma requires more than use of RD-Stage. It highlights the importance of cancer registries receiving administrative data from hospitals which captures the metastatic site (C77-C79) based on the ICD-10AM coding system [21]. The ICD-10AM has been developed and is maintained by the Australian National Centre for Classification in Health (NCCH). All Australian health services are required to collect for funding purposes the data field/s metastatic site, or the anatomical position (topography) of the secondary cancer. Yet, only in Victoria has this data been routinely collected across all tumour types and used by medical coders in capturing stage at diagnosis information [29]. If jurisdictions received this information from hospitals, they would be able to capture stage for nearly half of all patients diagnosed with pancreatic carcinoma.

The largest gap in capture of stage data is in those cases who have either local or regional disease and no accompanying pathology at diagnosis. It is likely that these cases were diagnosed using imaging data, which is not routinely available to cancer registries. Cancer Alliance Queensland was able to demonstrate that the addition of public imaging data, in combination with that captured from multidisciplinary team meeting notes and oncology systems enabled 76% of cases to be staged. Even with radiological data, it will not be possible to assign an N stage to many patients given that most do not have resection. There remains 1 in 4 cases currently unable to be staged even using these combined datasets. Imaging data is unlikely to be available in the short term, although access to this data could be a goal for cancer registries, as imaging will continue to make advances and having this information will make data collection more complete.

The RD-Stage concept of cancer registries using available data to classify T, N and M and derive stage/prognostic group has also been applied in the Essential TNM staging system, developed by a working group with representatives from the UICC, the International Agency for Research on Cancer and the International Association of Cancer Registries [30]. Essential stage coding guidelines were first published in 2019 for breast, cervix, colorectal and prostate cancer and have subsequently included lymphoma and cancers of the liver, oesophagus and ovary [31].

There are several limitations which impact the use of RD-Stage and the generalisability of findings. Firstly, as discussed, RD-Stage has no utility in jurisdictions not collecting the core elements of T, N and M. Secondly, RD-Stage (pancreatic carcinoma) validation was undertaken only in Victoria because other jurisdictions either did not have sufficient cases documenting stage (WA, SA, NT), used degree of spread assessments (NSW, ACT and Tas) or there was no validation set available (QLD). Future research would benefit from assessing the accuracy of RD-Stage (pancreatic carcinoma) calculated in Queensland where a large percentage of cases could be coded and the modified definition of M0 was used. Thirdly, RD-Stage calculation does not consider other prognostic factors such as biomarkers which are used to calculate stage in clinical practice and are incorporated into the most recent AJCC staging manual. For this reason, RD-Stage is not intended to replace stage collected in clinical practice. Finally, validation of RD-Stage was restricted to examining concordance on a small sample of 229 cases. While these cases were retrieved from the UGICR which captures data across both public and private hospitals, it may be that these hospitals contributing to UGICR have staging standards different from those who do not contribute to the registry.

This research emphasises the need for national effort to enhance capture of stage data. Standardisation of information collected by cancer registries should be a priority, and capture of ICD-10AM metastatic site data is low-hanging fruit, because it is already being coded by all Australian hospitals. The Queensland clinical advisory committee, responsible for prioritising data sources, identified multidisciplinary team meeting software as the prime source for high-quality stage data as it encompasses the assessment of all staging investigations by expert consensus of radiologists, surgeons, oncologists and pathologists [32]. Investment is needed to ensure that it is captured in such a way that it can be used by cancer registries with minimal manipulation. Future research could evaluate strategies to enable this information lifecycle to inform the development of a national multidisciplinary team meeting quality framework, with focus on record keeping of stage and treatment decisions. This project has demonstrated the importance of having standardised definitions to calculate stage and the complexity of attempting to develop rules when medical specialties use different staging systems, such as resectability. Research is required to develop rules for calculating stage across all tumour streams, expanding on those already developed for melanoma and cancers of the prostate, lung, breast, bowel [13] and endometrium [18].

Professional bodies such as the Royal College of Pathologists Australasia (RCPA) have introduced and now require for accreditation that pathologists use structured reporting protocols to standardise the capture of cancer information, including requirements for all tumours to be staged using the TNM classification system [33]. As laboratory information systems improve, this may even allow for discrete data elements within the reports to be standardised and tagged with ICD codes, allowing automatic transfer of the information to the cancer registries. Quality control through a robust audit process will be required to assess the completeness and accuracy of this and other collated information. While the RCPA states that TNM classification system should be the standard of care used in reporting pathological assessment of resections of pancreatic carcinoma, the Royal Australian and New Zealand College of Radiologists in their guidelines for writing reports state only that the report should include the precise anatomical location using accepted anatomical terminology and modality, the size or extent and other anatomical imaging characteristics relevant to diagnosis or treatment [34]. As such, radiology reports tend to describe the tumour in terms of resectability as this is the accepted terminology preferred by surgeons. Use of a standard reporting template for staging pancreatic cancer would ensure a complete description of the required characteristics and enable cancer registries to apply rules to derive resectability status. It is likely that both resectability and TNM staging will need to be collected to meet the needs of both the clinical community and those undertaking epidemiological studies. Cancer registries are exploring opportunities to expand the data sources from where information is available to stage cancer, and this should be fostered at a national level to enable benchmarking of cancer outcomes by stage of cancer at diagnosis. A national strategy is required to improve the quality of stage data recorded in data sources such as multidisciplinary team meeting software and medical records and thereby remove the resource-intensive process of deriving stage using rules.

As the most important prognostic factor associated with survival after a cancer diagnosis, without stage information we are unable to effectively examine variation in cancer care, the effectiveness of screening programs and the impact of public health interventions, such as those implemented over the previous two years to restrict spread of the COVID-19 virus. Accurate and consistent tumour stage classification is the cornerstone for the development and use of treatment guidelines. Treatment pathways exist to provide optimal care for patients at all stages of their disease [35], and it is as important that these are followed for patients with metastatic disease as for those with less advanced disease.

## Supporting information

S1 File Rules for staging pancreatic neoplasms. (PDF)

## References

[1] Denoix P. Nomenclature classification des cancers [in French]. Bull Inst Nat Hyg (Paris) 1952;7:743–8.

[2] Greene F, Sobin L. The Staging of Cancer: A Retrospective and Prospective Appraisal. CA: A cancer Journal for Clinicians. 2008;58(3):180–90. doi: 10.3322/CA.2008.0001 18460593

[3] Brierley JE, Gospodarowicz ME, Wittekind CE. TNM Classification of Malignant Tumours, 8th Edition: Wiley- Blackwell; 2016.

[4] Amin M, Edge S, Greene F, Byrd D, Brookland ReaE. AJCC Cancer Staging Manual, 8th edition. American Joint Commission on Cancer, editor. Chicago, IL: Springer International Publishing; 2017.

[5] Pecorelli S. Revised FIGO staging for carcinoma of the vulva, cervix, and endometrium. Int J Gynaecol Obstet. 2009;105(2):103–4. Epub 2009/04/16. doi: 10.1016/j.ijgo.2009.02.012 . 19367689

[6] National Cancer Institute. SEER Training Modules: Review of Staging Systems: NIH; [cited 2023 6 february]. Available from: https://training.seer.cancer.gov/collaborative/intro/systems_review.html.

[7] National Cancer Institute. SEER Training Modules: Code Structures. Examples of distinct bur similar appearing code structures NIH; [cited 2023 6 february]. Available from: https://training.seer.cancer.gov/operations/standards/setters/codes.html.

[8] Brierley J, O’Sullivan B, Asamura H, Byrd D, Huang SH, Lee A, et al. Global Consultation on Cancer Staging: promoting consistent understanding and use. Nat Rev Clin Oncol. 2019;16(12):763–71. Epub 2019/08/08. doi: 10.1038/s41571-019-0253-x ; PubMed Central PMCID: PMC7136160. 31388125 PMC7136160

[9] Hartwig W, Büchler MW. Pancreatic Cancer: Current Options for Diagnosis, Staging and Therapeutic Management. Gastrointest Tumors. 2013;1(1):41–52. Epub 2013/09/01. doi: 10.1159/000354992 ; PubMed Central PMCID: PMC4645567. 26673950 PMC4645567

[10] Lopez NE, Prendergast C, Lowy AM. Borderline resectable pancreatic cancer: definitions and management. World J Gastroenterol. 2014;20(31):10740–51. Epub 2014/08/26. doi: 10.3748/wjg.v20.i31.10740 ; PubMed Central PMCID: PMC4138454. 25152577 PMC4138454

[11] Cabasag CJ, Arnold M, Piñeros M, Morgan E, Brierley J, Hofferkamp J, et al. Population-based cancer staging for oesophageal, gastric, and pancreatic cancer 2012–2014: International Cancer Benchmarking Partnership SurvMark-2. Int J Cancer. 2021;149(6):1239–46. Epub 2021/05/16. doi: 10.1002/ijc.33679 . 33990959

[12] Tran TH, de Boer J, Gyorki DE, Krishnasamy M. Optimising the quality of multidisciplinary team meetings: A narrative review. Cancer Med. 2022;11(9):1965–71. Epub 2022/03/09. doi: 10.1002/cam4.4432 ; PubMed Central PMCID: PMC9089217. 35257515 PMC9089217

[13] Maharaj AD, Evans SM, Zalcberg JR, Ioannou LJ, Graco M, Croagh D, et al. Barriers and enablers to the implementation of multidisciplinary team meetings: a qualitative study using the theoretical domains framework. BMJ Qual Saf. 2021;30(10):792–803. Epub 2020/11/29. doi: 10.1136/bmjqs-2020-011793 . 33247002

[14] Cancer Australia. Stage, Treatment, and Recurrence (STaR) Strawberry Hills, NSW: Australian Government; [cited 2023 2nd April ]. Available from: https://www.canceraustralia.gov.au/research/data-and-statistics/cancer-data/improving-cancer-data.

[15] National Cancer Control Indicators. National Cancer Stage at Diagnosis Data Strawberry Hills, NSW: Cancer Australia; 2018 [cited 2022 23 December]. Available from: https://ncci.canceraustralia.gov.au/features/national-cancer-stage-diagnosis-data.

[16] Cancer Australia. National Cancer Control Indicators: distribution of cancer stage Strwaberry Hills, NSW: Australian Government; 2018 [cited 2023 2nd April]. Available from: https://ncci.canceraustralia.gov.au/diagnosis/distribution-cancer-stage/distribution-cancer-stage.

[17] Cancer Australia. National Cancer Control Indicators: 5-year relative survival from diagnosis Strawberry Hills, NSW: Australian Government; 2022 [cited 2023 2nd April]. Available from: https://ncci.canceraustralia.gov.au/outcomes/relative-survival-rate/5-year-relative-survival-diagnosis.

[18] Evans S, Ivanova K, Rome R, Cossio D, Pilgrim CHC, Zalcberg J, et al. Registry-derived stage (RD-Stage) for capturing cancer stage at diagnosis for endometrial cancer. BMC Cancer. 2023;In press. doi: 10.1186/s12885-023-11615-6 38087227 PMC10714535

[19] Cancer Australia. Capture of stage data Canberra, ACT2018 [updated 26th April 2018; cited 2023 3rd January]. Available from: https://ncci.canceraustralia.gov.au/diagnosis/distribution-cancer-stage/distribution-cancer-stage.

[20] National Comprehensive Cancer Consortium. NCCN Clinical Practice Guidelines in Oncology: Pancreatic Adenocarcinoma. 2022.

[21] World Health Organization (with Australian modifications developed by the National Centre for Classification in Health). International Statistical Classification of Diseases and Related Health Problems, Tenth Revision, Australian Modification (ICD-10AM). Darlinghurst, NSW: Independent Hospital Pricing Authority; 1998.

[22] Office of the Queensland Parliamentary Counsel. Hospital and Health Boards Act 2011. City East, Brisbane: 2017.

[23] Maharaj AD, Holland JF, Scarborough RO, Evans SM, Ioannou LJ, Brown W, et al. The Upper Gastrointestinal Cancer Registry (UGICR): a clinical quality registry to monitor and improve care in upper gastrointestinal cancers. BMJ Open. 2019;9(9). 10.1136/bmjopen-2019-031434. PubMed PMID: 2299399824. PMC6773358 31575580

[24] Yadav S, Sharma P, Zakalik D. Comparison of Demographics, Tumor Characteristics, and Survival Between Pancreatic Adenocarcinomas and Pancreatic Neuroendocrine Tumors: A Population-based Study. Am J Clin Oncol. 2018;41(5):485–91. Epub 2016/06/21. doi: 10.1097/COC.0000000000000305 . 27322698

[25] Farrukh J, Balasubramaniam R, James A, Wadhwani SS, Albazaz R. Pancreatic adenocarcinoma: imaging techniques for diagnosis and management. British journal of hospital medicine (London, England: 2005). 2022;83(5):1–12. doi: 10.12968/hmed.2022.0065 35653327

[26] Tempero MA, Malafa MP, Al-Hawary M, Behrman SW, Benson AB, Cardin DB, et al. Pancreatic Adenocarcinoma, Version 2.2021, NCCN Clinical Practice Guidelines in Oncology. Journal of the National Comprehensive Cancer Network: JNCCN. 2021;19(4):439–57. 10.6004/jnccn.2021.0017. 33845462

[27] Landis JR, Koch GG. The measurement of observer agreement for categorical data. Biometrics. 1977;33(1):159–74. Epub 1977/03/01. PubMed PMID: 843571. 843571

[28] Victorian Cancer Registry. E-Path Project Melbourne, Victoria2021 [cited 2021 3rd September ]. Available from: https://www.cancervic.org.au/research/vcr/recruitment-and-research-studies/e-path.

[29] Victorian Cancer Registry. Cancer Registration User Guide Melbourne, Victoria: Cancer Council Victoria; 2023 [cited 2023 8th Oct]. Available from: https://www.cancervic.org.au/research/vcr/submitting-data.

[30] Piñeros M, Parkin DM, Ward K, Chokunonga E, Ervik M, Farrugia H, et al. Essential TNM: a registry tool to reduce gaps in cancer staging information. Lancet Oncol. 2019;20(2):e103–e11. Epub 2019/02/05. doi: 10.1016/S1470-2045(18)30897-0 . 30712797

[31] International Agency for Research on Cancer (IARC) and at the Union for International Cancer Control (UICC). User’s Guide to Essential TNM (E TNM) 2022 [cited 3_8 Cancer Sites (April 2022)]. Available from: https://www.uicc.org/sites/default/files/atoms/files/Essential%20TNM%20Users%20Guide_Version%203_8%20sites%201%20Apr%202022_ENG.pdf.

[32] Soukup T, Lamb BW, Arora S, Darzi A, Sevdalis N, Green JS. Successful strategies in implementing a multidisciplinary team working in the care of patients with cancer: an overview and synthesis of the available literature. J Multidiscip Healthc. 2018;11:49–61. Epub 2018/02/07. doi: 10.2147/JMDH.S117945 ; PubMed Central PMCID: PMC5783021. 29403284 PMC5783021

[33] The Royal College of Pathologists of Australasia (RCPA). Postion Statement- Implementaiton of Structured Pathology Reporting of Cancer. Surry Hills, NSW: 2022.

[34] The Royal Australian and New Zealand College of Radiologists (RANZCR). Clinical Radiology Written Report Guidelines- Clinical Radiology, Version 7.0. Available at https://www.ranzcr.com/college/document-library/clinical-radiology-written-report-guidelines (accessed 2 March 2023): 2020.

[35] Cancer Council Victoria. Optimal care pathway for people with pancreatic cancer. Melbourne, Vic: 2021.

