# Supplementary material for: Registry-derived stage (RD-Stage) for capturing stage at diagnosis for pancreatic carcinoma in Australia
Source: PLoS One. 2024 Jan 2;19(1):e0294443. doi: 10.1371/journal.pone.0294443 (PMC10760927; doi:10.1371/journal.pone.0294443)

Supporting Information (S1): Rules for Staging Pancreatic Neoplasms

Evans SM, Ivanova K, Cossio D, Pilgrim CHC, Croagh D et al Registry-derived stage (RD-Stage) for capturing stage at diagnosis for pancreatic carcinoma in Australia. PLOS One: DOI: 10.1371/journal.pone.0294443

RULES FOR ASSIGNING TNM-M

The most common sites of distant metastasis for pancreatic carcinoma are liver, peritoneum, and lung. Metastasis to the bone, brain, and skin is uncommon.

More than half of the pancreatic carcinoma cases have distant metastasis at diagnosis.

Rules for assigning the M category for pancreatic carcinoma are outlined in Table 1.

Table 1: Rules for coding M- categories for pancreatic carcinoma

Value	Description	Rules	RD-Stage
**0**	No evidence of distant metastasis	Use "0" in cases with:<br>• no distant metastasis detected on imaging<br>• no distant metastasis reported on a hospital notification<br>• M=0 reported on medical records<br>• unknown, distant metastasis not stated	Record TNM-M=0 and continue to TNM-N
**1**	Distant metastasis	Use "1" in any of the following cases with reported as:<br>• distant metastasis on hospital notification<br>• distant metastasis on imaging<br>• distant metastasis on pathology such as:<br>– seeding to the peritoneum<br>– positive cytology from peritoneal washings<br>– metastasis to non-regional lymph nodes<br>– liver metastasis	**Record TNM M=1 and RD-Stage 4**<br><br>(Staging completed)

RULES FOR ASSIGNING TNM-N

Regional lymph nodes reported for pancreatic carcinoma are outlined in Table 2.

Table 2: Regional lymph nodes for pancreatic carcinoma

Tumour location	Regional Lymph nodes by tumour site	Defines TNM	ICD-10 code
Head and neck of the pancreas	Lymph nodes along:<br>• Common bile duct<br>• Common hepatic artery<br>• Portal vein<br>• Pyloric nodes<br>• Posterior and anterior pancreatoduodenal arcades<br>• Superior mesenteric vein<br>• Right lateral wall of the superior mesenteric artery	TNM N	C772

Body and tail of the pancreas	Lymph nodes along:<br>• Common hepatic artery<br>• Celiac axis<br>• Splenic artery<br>• Splenic hilum	TNM N	C772

The following rules should be used when assigning an N category:

i. Regional lymph nodes involved by direct tumour extension should be included in the count of positive lymph nodes

ii. Assessment of lymph node metastasis on imaging is based on:
    - nodal size on CT and MRI: lymph nodes >1 cm in the short axial dimension is considered abnormal
    - metabolic activity on PET/CT scan: metabolically active lymph nodes of any size are considered metastatic. PET/CT is considered superior imaging modality for assessing regional lymph node metastasis

Rules for each N-category are described below in Table 3.

Table 3: Rules for coding N- categories for pancreatic tumours

Value	Description	Rules	Staging basis	RD-Stage
**X**	Regional lymph nodes cannot be assessed	Use "X" in cases with:<br>• Unknown whether regional lymph nodes were assessed<br>Regional lymph nodes cannot be assessed	Clinical	Record TNM-N=X and continue to TNM-T
**0**	No regional lymph node metastasis	Use "0" in cases with reported:<br>• TNM N=0 in medical records and/or pathology<br>• no evidence of nodal involvement on imaging<br>• no metastasis identified in regional lymph nodes histologically	Clinical<br>Pathological	Record TNM-N=0 and continue to TNM-T
**1**	Metastasis in 1 to 3 regional lymph nodes	Use "1" in cases with reported:<br>• N=1 on medical report<br>• nodal metastasis as C772 on hospital notification without corresponding pathology<br>• metastasis to regional lymph nodes, number not specified<br>• metastasis in 1 to 3 regional lymph nodes on imaging and/or pathology<br>• Biopsy or FNA of positive regional node	Clinical/Pathological	**RD stage 2B** (stage completed)
**2**	Metastases in 4 or more regional lymph nodes	Use "2" in cases with reported:<br>• metastasis in 4 or more regional lymph nodes on imaging and/or pathology	Clinical/Pathological	**RD stage 3** (stage completed)

RULES FOR ASSIGNING TNM-T

Since 8th edition of AJCC staging classification the TNM T-value is not determined by the presence of direct tumour extension to adjacent organs such as:

- Ampulla of Vater
- Peritoneum
- Peripancreatic soft tissue
- Intrapancreatic part of the common biliary duct
- Stomach
- Spleen
- Left adrenal gland

Rules for classifying T-categories for pancreatic tumours are described in Table 5.

Table 5: Rules for coding T- categories for pancreatic tumours

Value	Description	Rules	Staging basis	RD-Stage
**X**	Unknown Primary tumour cannot be assessed	Use "X" in cases with:<br>• no information on primary tumour is available<br>• evidence of primary tumour, but no tumour size and/or extension reported on histology	Clinical<br>Pathological	Record TNM-T=X and continue to Table 9 to derive RD-Stage
**0**	No evidence of primary tumour	Use "0" in cases with reported:<br>• no residual tumour on imaging or pathology after Neoadjuvant treatment (NAT)<br>• complete response to NAT on imaging and/or pathology<br>Use "y0" in case of complete response to neoadjuvant therapy	Clinical/ Pathological	Record TNM-T=0 and continue to Table 9 to derive RD-Stage
**1**	Tumour ≤ 2cm greatest dimension	Use "1" in cases of tumour reported as:<br>• T=1<br>• minimally invasive tumour, size not specified	Clinical/ Pathological	Record TNM-T=1 and continue to Table 9 to derive RD-Stage
**1a**	Tumour ≤ 0.5 cm in greatest dimension	Use "1a" in cases of tumour reported as:<br>• T=1a<br>• tumour with size ≤5mm	Clinical/ Pathological	Record TNM-T=1a and continue to Table 9 to derive RD-Stage
**1b**	Tumour >0.5 cm and ≤1cm in greatest dimension	Use "1b" in cases of tumour reported as:<br>• T=1b<br>• tumour in size > 5mm and ≤10mm	Clinical/ Pathological	Record TNM-T=1b and continue to Table 9 to derive RD-Stage
**1c**	Tumour >1cm and ≤2cm	Use "1c" in cases of tumour reported as:<br>• T=1c<br>• tumour size greater than 10mm and ≤20mm	Clinical	Record TNM-T=1c and continue to Table 9 to derive RD-Stage

Value	Description	Rules	Staging basis	RD-Stage
**2**	Tumour >2cm and ≤ 4cm in greatest dimension	Use "2" in cases of tumour reported as:<br>• T=2<br>• Tumour size greater than 20mm and less or equal 40 mm	Clinical/ Pathological	Record TNM-T=2 and continue to Table 9 to derive RD-Stage
**3**	Tumour > 4cm in greatest dimension	Use "3" in cases of tumour reported as:<br>• T=3<br>• Tumour size greater than 40mm	Clinical	Record TNM-T=3 and continue to Table 9 to derive RD-Stage
**4**	Tumour involves celiac axis (CA), superior mesentery artery (CMA), and/or common hepatic artery (CHA), regardless of tumour size	Use "4" reported on medical records as:<br>• T=4 on imaging and/or medical records	Clinical	**RD stage 3**<br>(Stage completed)

CALCULATING PANCREATIC CARCINOMA STAGE FROM T-, N- and M- CATEGORIES

RD-Stage is an amalgamation of each of the T, N and M categories. Table 6 provides a summary of pancreatic carcinoma RD-Stage derived following coding of each category.

Table 6: R-D stage derivation from assigned T, N and M categories for pancreatic carcinoma.

RD-Stage	Tumour (t) category	Lymph node (N) category	Metastasis (M) category
**1A**	T1	N0	M0
**1B**	T2	N0	M0
**2A**	T3	N0	M0
**2B**	T1, T2, T3, TX	N1	M0
**3**	T1, T2, T3, TX	N2	M0
	T4	Any N	M0
**4**	Any T	Any N	M1
**9**	TX	N0, X	M0

USE OF RESECTABILITY AS A PROGNOSTIC INDICATOR IN PANCREATIC CANCER

Approximately 80-85% of all patients with pancreatic carcinoma have unresectable disease at the time of diagnosis. Therefore, imaging is the main type of investigation used to determine the stage at diagnosis for pancreatic carcinoma patients. The relationship of the tumour to blood vessels described on imaging reports is critical for determining the tumour resectablity status at multidisciplinary meetings.

High quality multi-phase imaging modalities enable clinicians to distinguish between patients eligible for tumour resection with curative intent and those with unresectable disease. A new clinical classification system was developed by the American Hepato-Pancreatico-Biliary Association and later adopted by National Comprehensive Cancer Network (NCCN), which classifies pancreatic carcinoma cases into the following categories:

- Resectable – technically resectable tumours with strong likelihood of achieving clear margins (complete surgical resection)
- Borderline resectable – technically resectable tumours, though often at the expense of vascular reconstruction, but with high risk of positive margins. These tumours can be eligible for Neoadjuvant treatment to improve the outcome of the following surgery.
- Unresectable
  - Locally advanced – tumours with low likelihood of resectability and high change of incomplete resection
  - Metastatic – presence of distant metastasis at diagnosis

Currently, there is no standard guideline that is accepted in Australia with agreed definitions of Resectable, Borderline Resectable, and Locally Advanced tumours. The Pancreatic carcinoma Expert Working Group agreed to use the NCCN guidelines, which states: "The decision regarding resectability status should be made by consensus at multidisciplinary meetings/discussions following the acquisition of dedicated pancreatic imaging including complete staging. Use of a radiology staging reporting template is preferred to ensure complete assessment and reporting of all imaging criteria essential for optimal staging, which will improve the decision-making process" This statement indicates two requirements:

- Imaging should be performed according to a staging protocol and reported using a template to ensure completeness, and

- The determination of resectability status requires the expertise of the multidisciplinary team, (rather than cancer registers directly relying on implicit language in the radiology report alone).

Therefore, the decision about diagnostic management and tumour resectability should be made by consensus at multidisciplinary meetings/discussions following appropriate high-quality imaging studies for evaluation the extent of disease. The definition of resectable, borderline resectable and unresectable pancreatic carcinoma according to the NCCN is described below in Table 4.

Table 4: Definition of resectable, borderline resectable and unresectable pancreatic carcinoma according to the National Comprehensive Cancer Network guidelines

**Type of involvement**	**Category**		
	**Resectable**	**Borderline resectable**	**Unresectable**
Vessel involvement<br>**SMV**- superior mesenteric vein<br>**PP** – Portal vein<br>**CA** - Celiac artery<br>**CHA** - Common hepatic artery<br>**SMA** - superior mesenteric artery<br>**IVC** - inferior Vena cava	• Without tumour - vessel contact<br><br>• Tumour contact of ≤180° (abutment) with SMV/PV **without** venous contour irregularity	**Veins:**<br>• Any type of tumour contact (abutment or encasement) **with** venous contour irregularity **OR** vein thrombosis, but **allowing** reconstructive surgery<br>• Tumour contact with IVC<br>**Arteries:**<br>• ≤180° tumour contact (abutment) with CA and/or SMA<br>• Tumour contact with CHA without extension to CA or hepatic artery bifurcation	• >180° tumour contact (encasement) with CA or SMA<br>• Tumour contact with aorta<br>• Tumour contact with the most proximal draining jejunal branch into SMV or the first jejunal SMA branch<br>• Unreconstractable SMV/PV due to tumour invasion or tumour thrombosis

Diagnostic and Treatment pathway for Pancreatic cancer

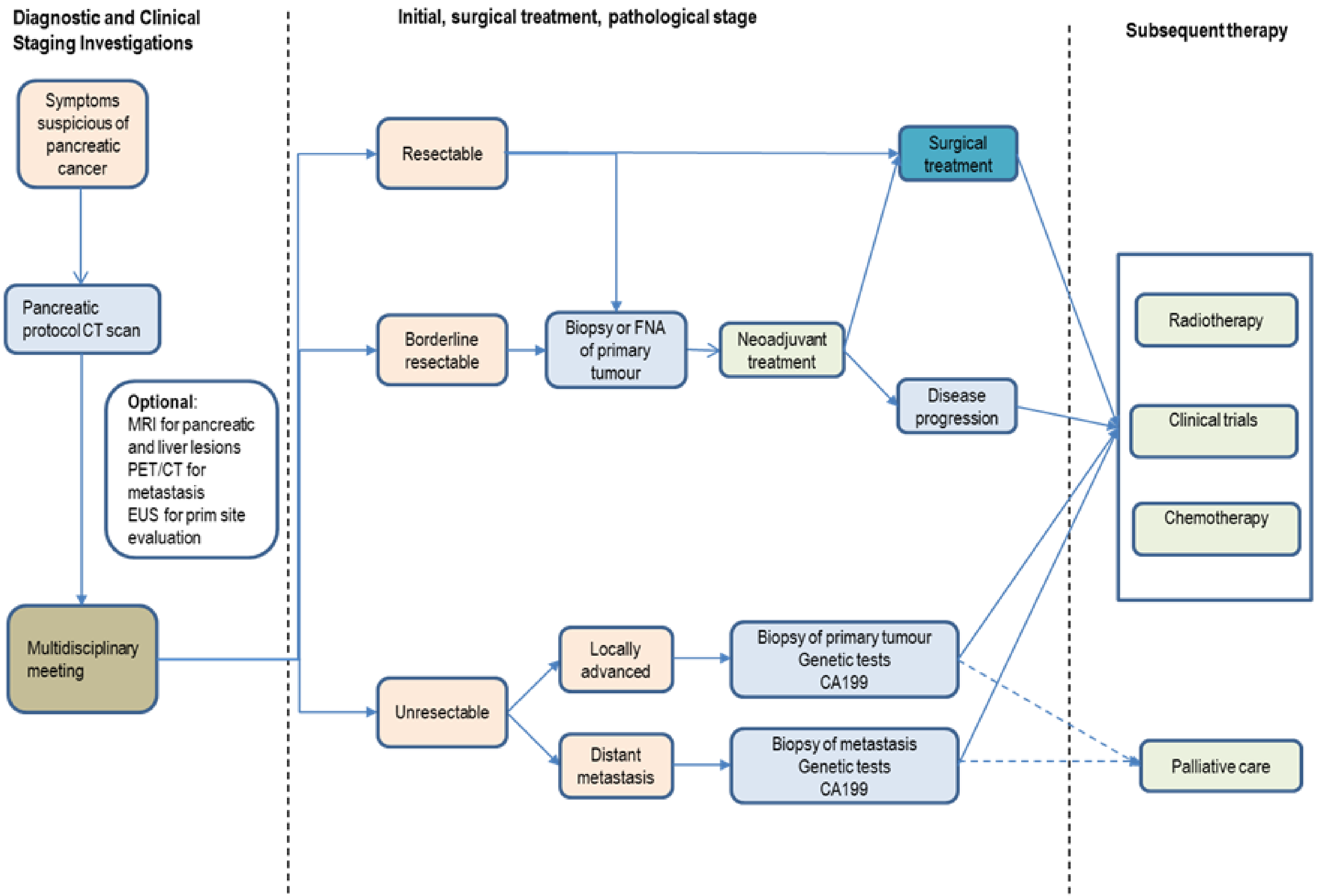

Supplement: S1 File — (PDF) [file pone.0294443.s001.pdf]
